# Angiotensin-Converting Enzyme and Renin-Inhibitory Activities of Protein Hydrolysates Produced by Alcalase Hydrolysis of Peanut Protein

**DOI:** 10.3390/ijms25137463

**Published:** 2024-07-07

**Authors:** Sukanya Poddar, Jianmei Yu

**Affiliations:** Food and Nutritional Sciences Program, Department of Family and Consumer Sciences, North Carolina Agricultural and Technical State University, Greensboro, NC 27411, USA; spoddar@aggies.ncat.edu

**Keywords:** peanut protein hydrolysate, Alcalase concentration, hydrolysis time, ACE inhibition, renin inhibition, Immunoglobulin E (IgE)-binding inhibition

## Abstract

Hypertension is a major controllable risk factor associated with cardiovascular disease (CVD) and overall mortality worldwide. Most people with hypertension must take medications that are effective in blood pressure management but cause many side effects. Thus, it is important to explore safer antihypertensive alternatives to regulate blood pressure. In this study, peanut protein concentrate (PPC) was hydrolyzed with 3–5% Alcalase for 3–10 h. The in vitro angiotensin-converting enzyme (ACE) and renin-inhibitory activities of the resulting peanut protein hydrolysate (PPH) samples and their fractions of different molecular weight ranges were determined as two measures of their antihypertensive potentials. The results show that the crude PPH produced at 4% Alcalase for 6 h of hydrolysis had the highest ACE-inhibitory activity with IC_50_ being 5.45 mg/mL. The PPH samples produced with 3–5% Alcalase hydrolysis for 6–8 h also displayed substantial renin-inhibitory activities, which is a great advantage over the animal protein-derived bioactive peptides or hydrolysate. Remarkably higher ACE- and renin-inhibitory activities were observed in fractions smaller than 5 kDa with IC_50_ being 0.85 and 1.78 mg/mL. Hence, the PPH and its small molecular fraction produced under proper Alcalase hydrolysis conditions have great potential to serve as a cost-effective anti-hypertensive ingredient for blood pressure management.

## 1. Introduction

Hypertension, a risk factor for both CVD and overall mortality [1,2], affected 31.1% of the global adult population (1.39 billion people) in 2010 [3]. Per the Centers for Disease Control (CDC), approximately 500,000 annual deaths in the United States can be attributed to high blood pressure [4]. Given its prevalence, lifestyle changes, dietary interventions, and pharmaceutical treatments are widely employed to address this condition [5]. The US CDC’s estimate suggests that the economic impact of hypertension in the United States ranges from USD 131 billion to USD 198 billion each year, encompassing healthcare expenses and productivity losses [4]. Renin and ACE play pivotal roles in the renin–angiotensin–aldosterone system (RAAS) pathway. Renin transforms angiotensinogen into angiotensin I (Ang I), which was further hydrolyzed by ACE to yield angiotensin II (Ang II), a potent vasoconstrictor. Ang II leads to vasoconstriction and prompts the release of aldosterone, elevating sodium levels and blood pressure [6]. Given this, ACE and renin inhibition stands as one of the strategies in hypertension treatment. Presently used synthetic drugs for hypertension management, such as captopril, alacepril, lisinopril, and enalapril, are mostly ACE inhibitors, and aliskiren is the only synthetic renin inhibitor approved by the FDA for managing hypertension [7]. The use of them causes undesirable side effects such as hypotension, cough, hyperkalemia, headache, dizziness, fatigue, nausea, and renal impairment, although they are effective [8]. Consequently, the exploration and development of safe and cost-effective antihypertensive compounds from food sources or the development of food therapy has garnered significant attention [9].

Various ACE-inhibitory peptides have been identified within diverse food protein sources like milk, fish, soybean, amaranth, and rapeseed [10,11,12,13,14]. Despite the limited understanding of the structure–function relationship of bioactive peptides, many of them share common attributes. Typically comprising 2 to 20 amino acids, these peptides tend to be rich in hydrophobic amino acids [15]. Numerous bioactive peptides derived from food have showcased their ability to counteract hypertension. These peptides often function by inhibiting ACE and decreasing renin activity [16,17]. Targeting renin alone within the Renin–Angiotensin System (RAS) does not completely prevent the breakdown of bradykinin catalyzed by ACE, which may result in vasoconstriction [18]. Consequently, a more effective strategy for reducing elevated blood pressure involves the development of natural therapeutic substances capable of producing diverse effects, such as concurrently inhibiting renin and ACE activities [19]. So far, the commercially available food-derived antihypertensive peptide products are mostly made from milk, fish, and algae [20]. No side effects have been reported for the intended use of food-derived bioactive peptides. Recently, peptides with renin-inhibiting activities have been found through the enzymatic breakdown of plant proteins produced from flaxseed and pea, opening new paths for research and potential therapeutic applications [21]. However, the study of antihypertensive properties of peptides and hydrolysates from peanuts is limited, mostly due to peanut allergy issues.

Peanuts, scientifically known as *Arachis hypogaea*, consist of approximately 50% lipid, primarily monounsaturated fat, 25% protein, and 8% dietary fiber, but minimal amounts of easily digestible carbohydrates like sugars and starches [22]. Due to the high oil content, peanuts are widely used to produce cooking oil in the world, which results in a large quantity of peanut flour/meal with a protein content of around 50%. Thus, peanut flour is a sustainable source of food protein and can be processed into peanut protein isolate (PPI) or PPC [23]. Limited studies show that protease hydrolysis of PPI and peanut flour also results in hydrolysates with certain ACE-inhibitory activities [22,24]. The production of antihypertensive peptides from food involves several steps: protein extraction, controlled hydrolysis, fractionation by ultrafiltration or preparative HPLC, and purification [20,25]. This process is complex, time-consuming, low yield, and high cost. The purpose of this study was to optimize the protease treatment condition to produce PPH with high ACE- and renin-inhibitory activity for possible food therapy of hypertension.

## 2. Results

### 2.1. Protein/Peptide and Amino Acid Concentrations of PPH

The PPC yield from peanut flour was 22.02 ± 1.41% (*n* = 9) under the extraction condition used in this study and the protein concentration of dried PPC was 80.39 ± 1.94% (*n* = 9). According to the product specification provided by the vendor, the protein content of peanut flour used in this study is 50 ± 2 g/100 g flour. Thus, the protein recovery of the extraction process was approximately 35.4%. The PPH was made by hydrolyzing 10% PPC at pH 8 and 40 °C. The highest protein concentration of 64 mg/mL was detected in the unhydrolyzed PPC solution, which means that only 79.61% of the protein in PPC was solubilized at the PH of enzymatic hydrolysis (pH 8). This is because of the lower protein solubility of peanut protein at pH 8 than at pH 10.

**Figure 1:**
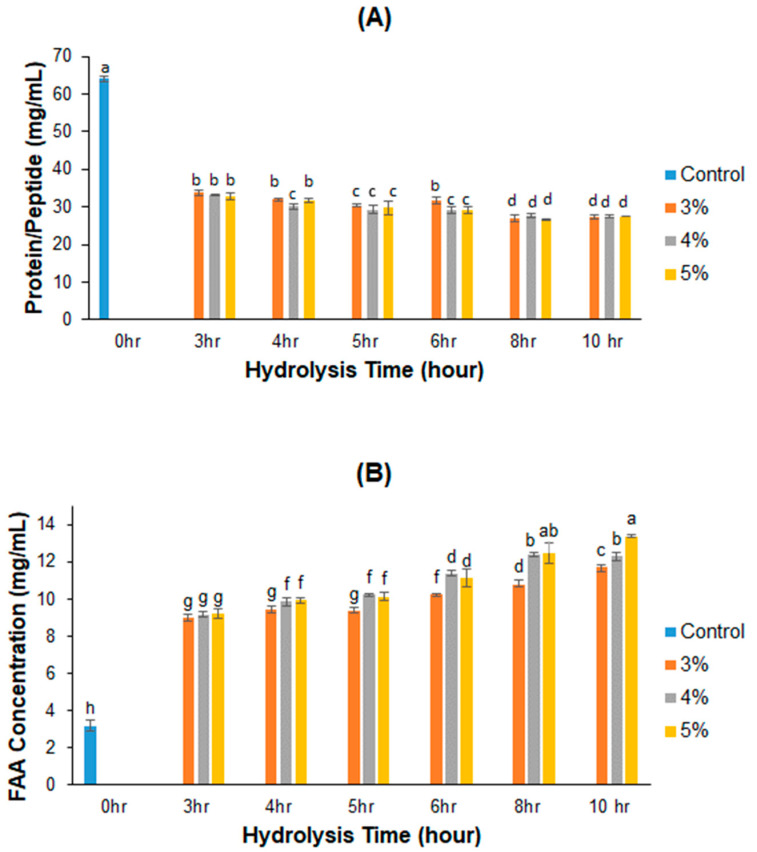
Effects of Alcalase concentration and hydrolysis time on the FAA and protein concentration of PPH. (**A**) Protein concentration, (**B**) FAA concentration (data bars with different labels represent significantly different values at *p* < 0.05).

As hydrolysis progresses, the protein concentration decreases due to the formation of peptides and amino acids, as shown by the gradually reduced protein concentration and steadily increased amount of free amino acids (FAA) with increasing hydrolysis time (Figure 1). At the same enzyme concentration, the protein/peptide concentration decreased logarithmically (R^2^ = 0.845–0.894) (Figure 1A), and the FAA increased linearly with treatment time (*p* < 0.0001, R^2^ = 0.896–0.987) (Figure 1B). Nevertheless, changes in protein concentrations with increasing Alcalase were small although significant in some cases at the same hydrolysis time. The protein concentration of the control sample, which did not undergo Alcalase treatment, was measured at 64.07 mg/mL. Among the treated samples, the highest protein concentration of 33.80 mg/mL was observed in the sample treated with a 3% Alcalase solution for a duration of 3 h. Conversely, the lowest protein concentration of 27.45 mg/mL was identified in the sample subjected to a 10 h hydrolysis at 3% Alcalase. Regarding the amino acid concentration, the control sample exhibited a minimum amino acid concentration of 3.19 mg/mL. Among the treated samples, the sample hydrolyzed for 3 h with 3% Alcalase demonstrated the lowest FAA concentration of 8.97 mg/mL, while the samples subjected to a 10 h treatment with 5% Alcalase peaked a FAA concentration of 13.38 mg/mL.

### 2.2. ACE-Inhibitory Activity (%) of Crude PPHs

**Figure 2:**
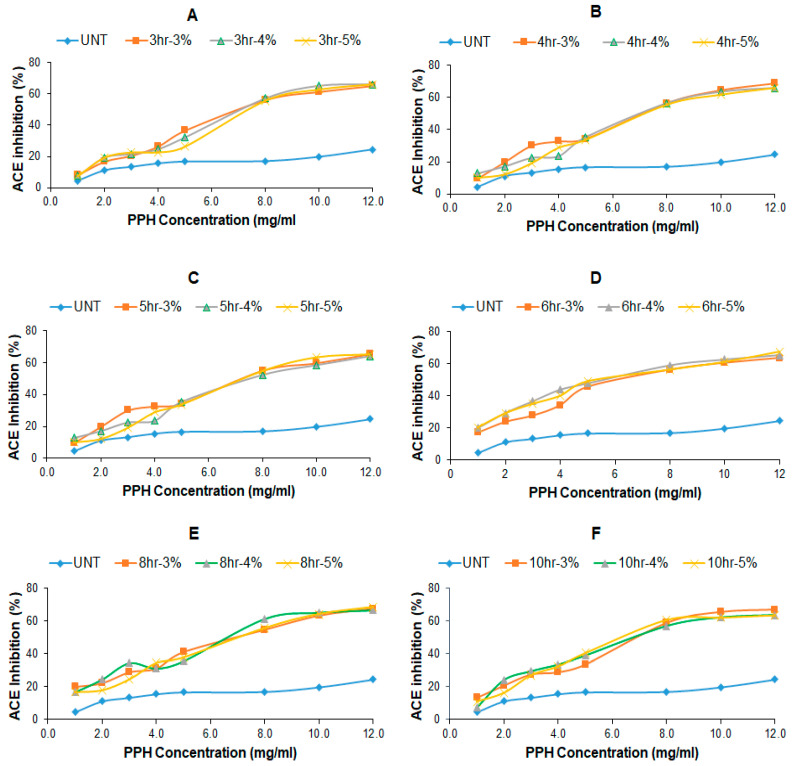
The sigmoid relationship between the ACE Inhibition (%) and concentration of crude PPH obtained at different hydrolysis times and Alcalase concentrations (%). UNT—untreated PPC, (**A**) 3 h, (**B**) 4 h, (**C**) 5 h, (**D**) 6 h, (**E**) 8 h, and (**F**) 10 h.

Alcalase hydrolysis of PPC resulted in hydrolysates with different degrees of ACE-inhibitory activities depending on hydrolysis time and Alcalase concentration. In this study, the ACE-inhibitory activity of PPC and PPH was assessed across concentrations ranging from 1 to 12 mg/mL. The percentages of ACE inhibition of both PPC and PPH increased with their concentration in a sigmoid pattern, as shown in Figure 2. The untreated PPC samples inhibited 4.4% to 24.5% ACE activity at a concentration of 1–12 mg/mL, while the percentage of ACE inhibition of PPH samples was in the range of 11.1–68.6%, greatly increased due to Alcalase hydrolysis. The IC_50_ values, the PPH concentrations required for 50% ACE inhibition, were determined and presented in Table 1. The smaller IC_50_ value corresponds to higher ACE-inhibitory potential. Notably, the lowest IC_50_ value was obtained for samples treated with different concentrations of Alcalase for 6 h. Specifically, the PPH obtained at 4% Alcalase over a 6 h hydrolysis period exhibited the lowest IC_50_ value of 5.45 mg/mL, indicating the highest ACE-inhibitory activity among tested samples. In contrast, samples subjected to 8 and 10 h of hydrolysis exhibited IC_50_ values lower than PPH samples treated for 3, 4, and 5 h but higher than those treated for 6 h. Table 1 also shows that the IC_50_ values of most PPH samples did not change significantly with Alcalase concentration at the same treatment time.

### 2.3. ACE-Inhibitory Activity (%) of Different Fractionations of PPH

This study evaluated the ACE-inhibitory activities of three fractions (F1: >10 kDa, F2: 5–10 kDa, and F3: <5 kDa) obtained from samples treated with different concentrations of Alcalase (3%, 4%, and 5%) for 6, 8, and 10 h because the crude PPH obtained under these hydrolysis conditions showed higher ACE-inhibitory activities. The ACE-inhibitory potential of these fractions was measured at protein/peptide concentrations of 0.5, 1.0, 1.5, and 2 mg/mL. Table 2 shows that the IC_50_ of various fractions of the same PPH sample decreased in the order of F1 > F2 > F3, indicating the increase in ACE-inhibitory activity of PPH with decreasing peptide size. Fraction 3 demonstrated the lowest IC_50_ values (0.85–1.68 mg/mL), which varied with the PPC hydrolysis condition. Among the different treatment conditions, samples treated for 6 h at Alcalase concentrations of 3, 4, and 5% displayed substantially higher ACE inhibition across all fractions (*p* < 0.05). The samples treated for 6 h with a 4% Alcalase exhibited the highest ACE inhibition, as evidenced by the lowest IC_50_ values across all fractions (0.87–3.68 mg/mL).

### 2.4. Renin-Inhibitory Activity of PPH

**Figure 3:**
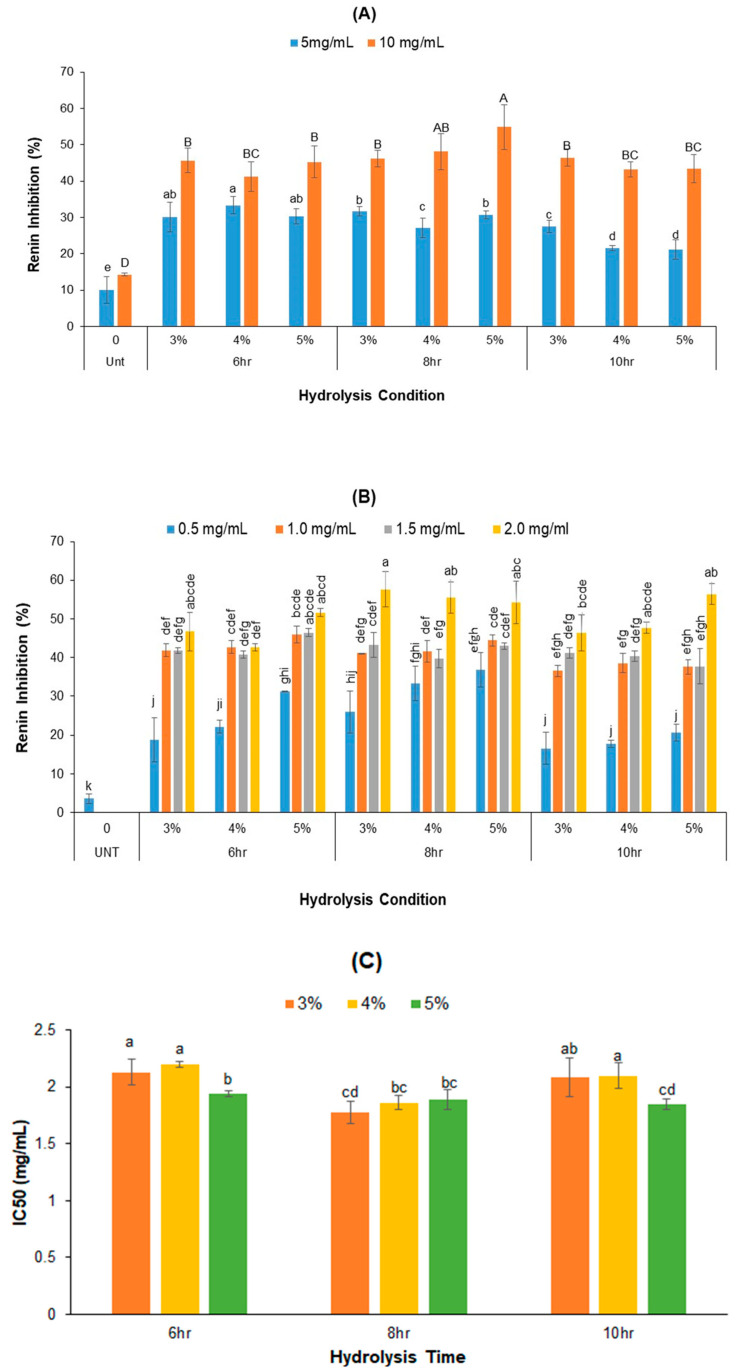
Effects of Alcalase concentration and hydrolysis time on the renin-inhibitory activity (%) of PPH. (**A**) Renin inhibition at the concentration 5 and 10 mg/mL of crude PPH (lowercase letters are for 5mg/mL, and uppercase letters are for 10 mg/mL), (**B**) renin-inhibitory activities of F3 of PPH samples at the different protein/peptides concentration, and (**C**) IC_50_ values of F3 samples (PPH fractions smaller than 5 kDa). (Data bars with different labels represent significantly different values at *p* < 0.05.)

In this study, the renin-inhibitory potential of crude PPH at concentrations of 5 and 10 mg/mL, alongside fractions smaller than 5 kDa (F3) at a protein/peptide’s concentration of 0.5, 1.0, 1.5, and 2.0 mg/mL, were assessed (Figure 3). Both the crude PPH and F3 demonstrated obviously higher renin inhibition compared to the control (PPC) (*p* < 0.05) across all concentrations (Figure 3A,B). Specifically, the crude PPH inhibited the renin activity by 17.08–32.56% at a concentration of 5 mg/mL and 41.25–54.88% at 10 mg/mL (Figure 3A), respectively. At the same concentrations, control samples only showed renin inhibitions of 10.03% and 17.59%, respectively. Figure 3A also demonstrated that (1) at PPH concentration 5 mg/mL, the renin-inhibitory activities of samples were in the order of 6 h hydrolysis > 8 h hydrolysis > 10 h hydrolysis, and (2) at PPH concentration of 10 mg/mL, the renin-inhibitory activities of samples obtained from 6 h and 10 h hydrolysis time were the same, but slightly lower than that of PPH samples obtained from 8 h hydrolysis. Notably, the crude PPH from 6 h hydrolysis with 4% Alcalase and the PPH from 8 h hydrolysis with 5% Alcalase exhibited the highest renin-inhibitory activity at 5 and 10 mg/mL, respectively.

Figure 3B shows that the smaller fraction of F3 of PPH displayed a renin inhibition rate of 16.57–36.80% at a concentration of 0.5 mg/mL, similar to crude PPH at 5 mg/mL but significantly higher than the F3 of control (untreated PPC). As total peptide concentration increased from 0.5 mg/mL to 1.0 mg/mL, the renin inhibition of F3 increased sharply. Further increase in F3 concentration from 1.0 mg/mL to 2 mg/mL resulted in a slight but significant increase in the renin inhibition with the exception of the samples hydrolyzed with 4% Alcalase for 6 h. It is worth mentioning that a significant renin inhibition of 54 to 57% was observed at a concentration of 2 mg/mL in all samples treated for 8 h (Figure 3B).

We also assessed the IC_50_ values of renin inhibition for the F3 samples (Figure 3C). The IC_50_ values ranged from 1.779 to 2.199 mg/mL. For the control sample, a low concentration of F3 (0.5 mg/mL) was used for the renin-inhibition test because the highest protein/peptide concentration in the F3 of the control sample was 0.5 mg/mL. Overall, the F3 samples from PPH treated for 8 h exhibited lower IC_50_ and the lowest IC_50_ was observed for samples treated for 8 h with a 3% Alcalase. The impact of Alcalase concentration on the renin-inhibitory activity of F3 is limited although the 6 h and 10 h hydrolysis with 5% Alcalase resulted in F3 having lower IC_50_ than other F3 samples under the same hydrolysis time.

### 2.5. IgE-Binding Inhibition of PPH

**Figure 4:**
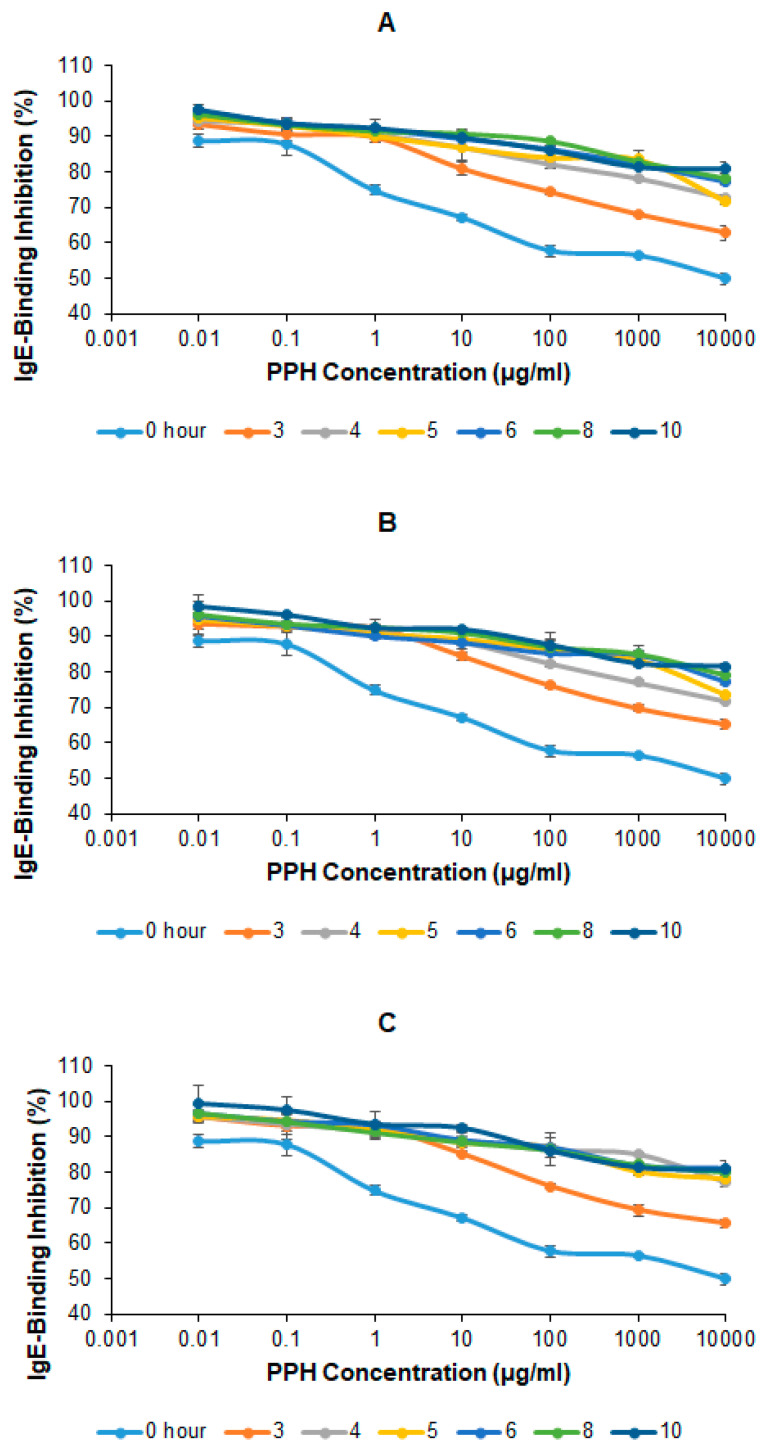
Percentages of IgE-binding inhibition of PPH samples produced at different Alcalase concentrations and hydrolysis time. (**A**) 3% Alcalase, (**B**) 4% Alcalase, and (**C**) 5% Alcalase.

This study also evaluated the *in vitro* allergenicity of PPH produced by extensive hydrolysis of PPC using a competitive inhibitory ELISA. Figure 4 shows that the IgE-binding inhibition of all crude PPH was higher than that of PPC. At all Alcalase concentrations, the percentage of IgE-binding inhibition of PPH increased with hydrolysis time, but no further increase was observed after 6 h of hydrolysis, which suggests the optimal hydrolysis time for reducing IgE binding of PPC at the Alcalase concentration tested in this study is 6 h. The IgE-binding inhibition of PPH samples produced by 6, 8, and 10 h hydrolysis at 100, 1000, and 10,000 µg/mL were 48–52%, 44–50%, and 55–63% higher than those of PPC, respectively, varying slightly with Alcalase concentration.

## 3. Discussion

The use of Alcalase to hydrolyze PPC resulted in a decrease in protein concentration but a consistent increase in the quantity of free amino acids (FAA) with time. The decrease in detectable soluble protein after hydrolysis can be explained by the following two reasons. First, the peptides turned into amino acids, which could not be measured using the BCA method due to the lack of peptide bonds. This is evidenced by the increase in free amino acids with the progress of hydrolysis. Secondly, hydrophobic peptides form aggregates and become insoluble in a water or buffer solution due to their nature [26]. Regarding the increase in the amino acid concentration, enzymatic hydrolysis by protease is a process by which proteins are broken down into peptides and amino acids [27]. The assessment of FAA production is a reliable method for evaluating the progress of the protein hydrolysis process [28]. Alcalase is a protease preparation dominated by subtilisin, which is an endopeptidase with broader substrate specificity than chymotrypsin, although they both have a catalytic triad of Asp, His, and Ser [29]. Depending on the position of Asp, His, and Ser, the end products can be peptides and amino acids. In the present study, the amino acid concentration of PPH increased significantly with both Alcalase concentration and hydrolysis time.

Our investigation found that the hydrolysis of PPC led to a considerable increase in ACE-inhibitory activity at various concentrations and treatments. The results suggest a significant dependence of ACE inhibition on the hydrolysis duration, rather than on the concentration of Alcalase when its concentration was 3% or higher. This is consistent with the findings of prior studies [22,24]. In an early study reported by Jamdar and colleagues [24], the hydrolyzed PPI exhibited 90 to 97% ACE inhibition depending on the degree of hydrolysis, while the unhydrolyzed PPI inhibited ACE by 66%, which was greatly higher than the ACE inhibition of unhydrolyzed plant proteins reported in other studies [22,30] and that of this study. In the study of Giromini et al. (2017), the ACE inhibitions of undigested and gastric-digested plant proteins were 8–36% and 32–81%, respectively [30]. Our previous study demonstrated that the ACE inhibition of untreated peanut protein extract was 3.8–4.7% at a protein concentration of 1 mg/mL, and it increased to 35–37% after 4 h of hydrolysis [22]. The different ACE sources might be the reason for the large difference between Jamdar’s study and other studies. In Jamder’s study, the ACE was self-prepared from swine lung, while the ACE used in our previous study and the current study was from rabbit lung and was purchased from Sigma-Aldrich.

It has been reported that the ACE-inhibitory activities of peptides are affected by the size of the peptide, and the smaller peptides often have stronger ACE-inhibitory activity [31,32]. The results of this study further confirmed that peptide size directly impacts ACE-inhibitory activity, with smaller peptides showing stronger inhibition as shown by the much smaller IC_50_ of F3 compared to the IC_50_ values of crude PPH, F1, and F2 (Table 1). Smaller peptides are more likely to bind to ACE’s active site, leading to enzyme inhibition [33]. The F3 obtained from PPH hydrolyzed for 6 h had IC_50_ value of 0.85–0.89 mg/mL, which is close to the IC_50_ of Captopril (0.5 mg/mL) and the 3 kDa permeate (0.59 mg/mL) of brown seaweed *Laminaria digitate* hydrolyzed by 1% (*v*/*w*) Viscozyme^®^ and Alcalase^®^, as reported in a recent study [34]. However, the quantity of fraction 3 was limited and its concentration remained relatively low (between 1.5 and 2 mg/mL). Thus, it is resource intensive and time consuming to produce Fraction 3. Thus, it is important to optimize the protease hydrolysis conditions to produce crude PPH with the highest ACE-inhibitory activity. Consequently, the consumption of PPH as a dietary supplement or food could offer blood pressure-lowering benefits for individuals with hypertension, at a relatively higher dose.

The ACE-inhibitory activity of a hydrolysate can be influenced by both the duration of hydrolysis and the concentration of protease employed. Deviating from the optimal hydrolysis time, either by making it longer or shorter, can result in reduced or diminished ACE inhibition. This occurs because shorter hydrolysis times may fail to produce sufficient effective peptides, whereas longer hydrolysis times may break down effective peptides into smaller, less potent peptides or free amino acids [32]. The ideal hydrolysis conditions depend on the specific protein source and the type of protease utilized [33]. In this study, the hydrolysis time of 6 h at an Alcalase concentration of 4% (*v*/*w*) resulted in crude PPH and diffractions with the lowest IC_50_ values, that is, the highest ACE-inhibitory activities. The lower percentages of ACE inhibition of fractions obtained at 8 and 10 h of enzymatic treatment compared to those derived from the 6 h samples is most likely due to the degradation of some ACE-inhibitory peptides into FAA as evidenced by the significantly lower protein/peptide concentration and higher FAA concentration in the PPH (Figure 1).

Renin inhibitors provide precise inhibition within RAS, yielding improved therapeutic profiles by selectively targeting renin-catalyzed hydrolysis of angiotensinogen [35]. Compared to ACE inhibition, there have been limited studies concerning the renin-inhibitory characteristics of peptides derived from food proteins. The slower progress in this domain might be attributed to the greater challenge in renin inhibition assay, coupled with the notably higher expenses associated with renin assay procedures compared to those for ACE [25]. Only a few studies reported the renin-inhibitory abilities of some food protein hydrolysates, including hemp seed, rapeseed, canola seed, macroalga, kidney bean, and African yam seed [36,37,38,39,40,41]. A common finding of the above studies is that the crude protein hydrolysate displayed higher ACE and renin-inhibitory activity than the smaller weight fractions. In contrast to the above studies, the small molecular fraction of PPH showed higher ACE and renin-inhibitory activity than crude PPH in this study. This might be due to the difference in protein source and protease used for protein hydrolysis. It was reported that the PPH produced by hydrolysis of microfluidized peanut protein using the combined Neutrase and Protamex contained peptides mostly smaller than 1 kDa and displayed 77% of renin-inhibitory activity at 2 mg/mL [42], which is higher than the crude PPH produced by direct Alcalase hydrolysis and comparable to the F3 in this study. The studies summarized in a recent review show that the rank of the renin-inhibitory activities of protein hydrolysates from different protein sources is oil seeds (including canola, rape seeds, hemp seeds, and peanuts) ≥ pea > chicken meats > beans > bovine proteins [43]. Among protein hydrolysates of animal origin, fish protein hydrolysates exhibited the highest renin inhibition, while bovine protein hydrolysates showed negative or no renin inhibition compared to unhydrolyzed bovine proteins, although they were reported to have significant ACE-inhibitory activity both in vitro and in vivo [43]. This suggests that the plant-protein hydrolysates may be more effective antihypertensive ingredients, and thus function better than animal-protein hydrolysates in blood pressure control.

Research shows that about 2% of the Western population is affected by peanut allergy, with 7–14% of cases caused by accidental peanut exposure annually, and one-third to one-half may experience anaphylaxis [44]. As a result, studies on using peanut protein to produce dietary supplements and nutraceuticals are limited. Peanut allergy is an IgE-mediated food allergy that occurs when patients develop IgE antibodies against the peanut proteins, followed by exposure to that protein [45]. This study shows that the IgE binding of peanut protein was reduced by 48–63% after 5 h of hydrolysis by Alcalase, which was consistent with the skin-prick test results from a previous study [31] and confirmed what was stated in a recent study that longer than 4 h of hydrolysis duration was needed to further reduce the allergenicity of peanut protein [22]. The significant reduction of IgE binding indicates the formation of non-allergenic peptides during PPC hydrolysis. Due to the remaining allergenicity, allergen labeling is still needed for the products containing antihypertensive crude PPH.

## 4. Materials and Methods

### 4.1. Materials

The light roasted defatted peanut flour (containing 12% lipid and 50 ± 2% protein) was purchased from the Golden Peanut Company (Albany, GA, USA). The Novozyme 2.4 L of Alcalase, leucine, ninhydrin reagent, ACE (0.5 U/mL) derived from rabbit lung, an enzyme substrate known as FAPGG peptide, goat anti-human IgE peroxidase conjugate, and o-phenylenediamine were obtained from Sigma-Aldrich (St. Louis, MO, USA). Sodium hydroxide, Bicinchoninic acid (BCA) reagents, Tris base, Tween 20, 30% hydrogen peroxide, and other chemicals were acquired from Fisher Scientific (Waltham, MA, USA). Renin-inhibitory Screen Kits were purchased from Cayman Chemical Company (Ann Arbor, MI, USA).

### 4.2. Preparation of Peanut Protein Concentrate

PPC was produced using the isoelectric precipitation method, following a modified version of the procedure described by [23]. Briefly, 100 g of defatted light roasted peanut flour was mixed with 900 mL of deionized (DI) water at a 1:10 ratio (*w*/*v*). The pH of the mixture was then adjusted to 10.0 using a 2N solution of sodium hydroxide (NaOH). The resulting suspension of peanut flour was agitated at 35 °C and 250 rpm for 1 h in a water bath shaker. Subsequently, the suspension was centrifuged at 3000× *g* for 20 min to separate the solid and liquid components. The supernatant, the liquid portion, was collected and its pH was adjusted to 4.8–5.0 using a 2N solution of hydrochloric acid (HCl). Following this, the suspension was centrifuged again at 3000× *g* for 20 min. The supernatant was discarded, and the remaining precipitate was collected and freeze-dried at −5 °C to obtain the final PPC product. The protein content of dry PPC was determined by a nitrogen analysis method using a 2400 CHN Elemental Analyzer (PerkinElmer, Waltham, MA, USA) and a nitrogen-to-protein conversion factor of 5.46 [46].

### 4.3. Preparation of Peanut Protein Hydrolysate (PPH)

A 3 × 6 two-factor factorial design was used to optimize the PPH production condition. The protease hydrolysis was conducted with 3, 4, and 5% Alcalase for 3, 4, 5, 6, 8, and 10 h at pH 8 and 40 °C. The process involved suspending 1.00 g of PPC in 10 mL of phosphate buffer solution. The pH of the mixture was adjusted to 8.0 using a 1N NaOH solution. Subsequently, the PPC suspension was incubated with different concentrations (3, 4, and 5%) of 2.4 L Alcalase for a duration of 3 to 10 h in a water bath shaker at 40 °C and 200 rpm. The pH of the solution was maintained at 8.0 throughout the enzymatic hydrolysis process by adding 2N NaOH solution as necessary. Control samples were prepared by incubating the peanut flour suspensions without the addition of Alcalase. At specific time points, samples were taken and immediately subjected to enzyme inactivation by placing them in a 90 °C water bath for 10 min. Thereafter, the samples were cooled in ice water and then centrifuged at 3000× *g* for 20 min. The resulting supernatants (PPH) were collected, divided into small quantities, and stored at −20 °C for further utilization.

### 4.4. Fractionation of Peanut Protein Hydrolysates

To obtain PPH fractions with different ranges of molecular weights, the PPH was diluted with phosphate buffer at a ratio of 1:7 and subjected to sequential centrifugation using ultrafiltration (UF) centrifugal tubes of molecular weight cut-off (10 kDa and 5 kDa). This process resulted in three fractions with a molecular weight greater than 10 kDa (Fraction 1), within 10 to 5 kDa (Fraction 2), and smaller than 5 kDa (Fraction 3). Each fraction was split into small portions and stored at −20 °C until use.

### 4.5. Determination of Protein/Peptide and Amino Acid Concentrations in PPC and PPH

The protein/peptide concentrations in the samples were measured by the Bicinchoninic Acid (BCA) method, following the microplate procedure using a Synergy HTX Microplate Reader (Bio Tek, Winooski, VT, USA). Bovine serum albumin (BSA) solution (ThermoFisher Scientific, High Point, NC, USA) was employed as the standard. The protein concentration of each sample was determined in triplicate. FAA concentrations in PPC and PPH samples were determined by the ninhydrin method using a 2% ninhydrin reagent solution purchased from Millipore Sigma and leucine standard solutions according to the manufacturer’s instructions [47].

### 4.6. ACE-Inhibitory Activity Assay

The ACE-inhibitory activity was assessed following a previously described method [9]. In brief, 10 μL of ACE solution (0.5 U/mL, from rabbit lung) and 10 μL of either PPH/fractions of PPH or demineralized water (used as a blank) were added to the wells of a 96-well microplate. The reaction was initiated by adding 150 μL of prewarmed substrate solution containing FAPGG (1 mM in 50 mM Tris-HCl with 0.3 M NaCl, pH 7.5) to each well. The microplate was then incubated at 37 °C, and the absorbance at 340 nm was recorded every minute for a duration of 30 min using an HTX Microplate Reader using kinetic mode. The slope of the linear portion of the absorbance vs. reaction time curve was utilized as a measure for the calculation of ACE-inhibitory activity according to the provided equation:

ACE inhibition (%) = [1 − (ΔA_Inhibitor_/ΔA_Control_)] × 100

where ΔA_Inhibitor_ is the slope of the sample with the inhibitor and ΔA_Control_ is the slope of the control. Each experiment was performed in three replicates. Using non-linear regression analysis of ACE-inhibitory activity (%) versus peptide concentration, the IC_50_ value or the concentration of PPH/fractions of PPH needed to generate 50% ACE inhibition under the conditions specified was calculated.

### 4.7. Renin Inhibition Assay

The inhibition of human recombinant renin activity was assessed using the Renin Inhibitor Screening Assay Kit, following previously established protocols [35]. Before conducting the assay, the renin buffer was diluted 10 times with Milli-Q water and the renin enzyme solution was diluted 20 times with assay buffer prior to use, and the assay buffer was pre-warmed to 37 °C before the reaction was started. Prior to the reaction, specific amounts of substrate, assay buffer, and Milli-Q water were added to different wells. For the assay setup, the initial activity wells contained 20 μL substrate, 160 μL assay buffer, and 10 μL Milli-Q water. Background wells were prepared with 20 μL substrate, 150 μL assay buffer, and 10 μL Milli-Q water, while inhibitor wells contained 20 μL substrate, 150 μL assay buffer, and 10 μL sample. Subsequently, the reaction was started by adding 10 μL renin to the control and sample wells. Following a 10 s shaking step for thorough mixing, the microplate was then incubated at 37 °C, and the fluorescence intensity (FI) was measured using excitation and emission wavelengths of 340 nm and 490 nm, respectively, every minute for a duration of 30 min using the HTX Microplate Reader (Bio Tek, Winooski, VT, USA) using kinetic mode. The percentage inhibition was computed using the following formula:

Renin inhibition (%) = [1 − (ΔFI_Inhibitor_/ΔFI _Control_)] × 100

where ΔFI_Inhibitor_ is the slope of the sample with the inhibitor and ΔFI_Control_ is the slope of the control. Each experiment was performed in three replicates.

### 4.8. Assessing the IgE Binding of PPH Using Human Serum

In accordance with previously established protocols [9], a competitive inhibition enzyme-linked immunosorbent assay (ciELISA) was conducted to determine the IgE binding of PPH at concentrations of 0–10,000 µg/mL. A microplate was coated with untreated PPC, which was diluted at a ratio of 1:20 with PBS and incubated at 37 °C for 2 h. Following three washes with PBST (pH 7.4), the plate was subjected to a three-blocking step with 200 µL of 1% BSA-PBST at room temperature, each 5 min. The plate was washed three times after each blocking. After this, 50 µL of PPC or PPH sample was mixed with 50 µL of 1:50 diluted pooled human serum obtained from 7 peanut-allergic patients (Lab Plasma International Inc., Everett, WA, USA) and added to respective wells. Subsequently, the plate was gently shaken for 45 min at room temperature, followed by three washes with PBST. The presence of immunoglobulin E (IgE) antibodies bound to the plate was then detected using a goat anti-human IgE peroxidase conjugate (Sigma-Aldrich, St. Louis, MO, USA) (diluted at 1:1000, 100 μL) and a substrate solution (100 µL) containing o-phenylenediamine (0.5 mg/mL) and 0.03% hydrogen peroxide in 0.1 M citrate buffer (pH 5.5). The enzymatic reaction was halted with 2 N sulfuric acid (50 µL), and the absorbance was measured at 490 nm using an HTX Microplate Reader. The absorbance of the sample containing both IgE and peanut sample was denoted as B, while B_0_ represented the absorbance of a control comprising IgE alone. Notably, the higher the allergenicity of the sample, the lower the absorbance, and conversely, as previously described [48]. The degree of IgE binding inhibition within a sample was quantified as B × 100/B_0_.

### 4.9. Statistical Analysis

Changes in protein and FAA concentrations during hydrolysis were analyzed using linear regression and post-ANOVA Tukey tests at a significance level of 5%. Additionally, IC_50_ values for the ACE and renin-inhibitory activities of crude PPH and the fractions of PPH were determined across various hydrolysis conditions using a non-linear regression curve. To evaluate distinctions among IC_50_ values of different samples, post-ANOVA Tukey tests were conducted at a significance level of 5%. Regression analysis and Tukey tests were conducted using GraphPad Prism version 8.0.2 (263) (GraphPad Software, Boston, MA, USA).

## 5. Conclusions

This study revealed that the hydrolysis of PPC with Alcalase under optimal conditions could result in crude PPH with moderate capacity to inhibit the activities of ACE and renin, two critical enzymes in blood pressure regulation. This is an important advantage over the protein hydrolysates/peptides derived from animal proteins, except for fish proteins. Since renin catalyzes the rate-determining step in the renin–angiotensin–aldosterone system, the protein hydrolysates with both renin and ACE-inhibitory activities and should work better in blood pressure attenuation. The optimal Alcalase concentration and hydrolysis time for high ACE-inhibitory PPH were 4% and 6 h, respectively, different from that for high renin-inhibitory PPH (5% Alcalase and 8 h hydrolysis time). Within the spectrum of PPH fractions, F3 (<5 kDa) consistently showcased higher ACE-and renin-inhibitory activities regardless of the Alcalase concentration or hydrolysis time, but its availability was limited because of the low yield from fractionation and low concentration (1.5–2 mg/mL). Thus, the production of F3 could be both costly and time-consuming. Consequently, crude PPH holds promise as a potential dietary supplement or functional food for blood pressure regulation among individuals with hypertension. In addition, the crude PPH showed significantly reduced IgE binding, which implicates lower allergenicity, compared to untreated PPC. However, hydrolysis of plant protein has been reported to generate bitter peptides [49,50]; thus, it is important to evaluate the sensory properties of PPH and explore practical methods to eliminate or mask the bitterness without reducing its antihypertensive potential need to be studied. To establish the therapeutic effects of PPH- or PPH-enriched foods on blood pressure control, further investigations encompassing in vivo hypertension studies and human clinical trials are needed.

## Figures and Tables

**Table 1:** IC_50_ (mg/mL) for ACE inhibition of crude PPHs produced at different Alcalase concentrations and hydrolysis times.

Alcalase Concentration	Hydrolysis Time (h)					
	3 h	4 h	5 h	6 h	8 h	10 h
**3%**	6.94 ± 0.19 ^b^	6.89 ± 0.13 ^b^	6.63 ± 0.02 ^a^	6.36 ± 0.08 ^c^	6.84 ± 0.02 ^b^	6.67 ± 0.22 ^b^
**4%**	6.78 ± 0.34 ^b^	6.73 ± 0.14 ^b^	6.13 ± 0.09 ^a^	5.45 ± 0.20 ^d^	6.37 ± 0.06 ^c^	6.46 ± 0.20 ^c^
**5%**	7.40 ± 0.30 ^a^	7.05 ± 0.06 ^b^	6.34 ± 0.24 ^a^	5.93 ± 0.37 ^d^	6.78 ± 0.31 ^c^	6.17 ± 0.14 ^d^

Note: in the same row, data with different superscripts are significantly different at *p* < 0.05.

**Table 2:** IC_50_ (mg/mL) values for ACE inhibition of different fractions of PPHs produced at different Alcalase concentrations and hydrolysis times.

Hydrolysis Time	Alcalase Concentration	IC_50_ (mg/mL)		
		Fraction 1	Fraction 2	Fraction 3
	3%	5.43 ± 0.49 ^c^	3.38 ± 0.30 ^b^	0.89 ± 0.02 ^e^
**6 h**	4%	3.68 ± 1.09 ^d^	1.57 ± 0.11 ^d^	0.87 ± 0.05 ^e^
	5%	5.75 ± 1.22 ^c^	3.56 ± 0.29 ^b^	0.85 ± 0.01 ^e^
	3%	7.35 ± 0.20 ^a^	5.23 ± 1.07 ^c^	1.50 ± 0.01 ^bc^
**8 h**	4%	5.23 ± 1.27 ^c^	4.34 ± 0.23 ^bc^	1.56 ± 0.06 ^bc^
	5%	6.51 ± 0.22 ^bc^	4.30 ± 0.66 ^bc^	1.68 ± 0.03 ^a^
	3%	5.76 ± 0.13 ^c^	5.39 ± 0.04 ^ac^	1.59 ± 0.05 ^ab^
**10 h**	4%	4.03 ± 0.06 ^d^	3.34 ± 0.59 ^b^	1.47 ± 0.01 ^c^
	5%	5.90 ± 0.09 ^c^	4.39 ± 0.02 ^bc^	1.36 ± 0.04 ^d^

Note: in the same column, data with different superscripts are significantly different at *p* < 0.05.

## Data Availability

Data will be made available on request.
